# Fluorescence Cholangiography for Extrahepatic Bile Duct Visualization in Urgent Mild and Moderate Acute Cholecystitis Patients Undergoing Laparoscopic Cholecystectomy: A Prospective Pilot Study

**DOI:** 10.3390/jcm14020541

**Published:** 2025-01-16

**Authors:** Janis Pavulans, Nityanand Jain, Kaspars Zeiza, Elza Sondore, Krista Brigita Cerpakovska, Janis Opincans, Kristaps Atstupens, Haralds Plaudis

**Affiliations:** 1 Department of Surgery, Riga Stradinš University, 16 Dzirciema Street, LV-1007 Riga, Latvia; kaspars.zeiza@rsu.lv (K.Z.); janis.opincans@rsu.lv (J.O.); hplaudis@gmail.com (H.P.); 2 Department of Doctoral Studies, Riga Stradinš University, 16 Dzirciema Street, LV-1007 Riga, Latvia; 3 Department of General Surgery, Riga East Clinical University Hospital, LV-1038 Riga, Latvia; 017299@rsu.edu.lv (E.S.); kc13040@students.lu.lv (K.B.C.); kristofss@inbox.lv (K.A.); 4 Statistics Unit, Riga Stradinš University, 16 Dzirciema Street, LV-1007 Riga, Latvia; nityapkl@gmail.com

**Keywords:** gallstone disease, laparoscopic cholecystectomy, fluorescence cholangiography, critical view of safety, acute cholecystitis

## Abstract

**Background:** Laparoscopic cholecystectomy for acute cholecystitis carries an increased risk of biliovascular injuries. Fluorescence cholangiography (FC) is a valuable diagnostic tool for identifying extrahepatic bile ducts (EHBD). The objective of this study was to evaluate the efficacy of FC in delineating EHBD anatomy, both before and after dissection, based on the critical view of safety (CVS) principles. **Methods:** Urgently admitted patients were prospectively stratified into two groups, depending on whether they had mild or moderate acute cholecystitis, in accordance with the 2018 Tokyo guidelines. All patients were scheduled for an early laparoscopic cholecystectomy using FC and were administered a fixed dose of indocyanine green (ICG) intravenously 12 h prior to the surgical procedure. **Results:** A total of 108 patients—75 patients with mild acute cholecystitis and 33 patients with moderate acute cholecystitis—were included. More than four CVS steps were performed in 101 patients (93.5%). Less than four CVS steps were performed only in seven patients—three (2.5%) patients with mild acute cholecystitis and four (4%) patients with moderate acute cholecystitis. The achievement of the CVS principles and the visualization rate using FC significantly increased in both patient groups, ranging from 3% before CVS to 100% after CVS (*p* < 0.001). In both groups, the cystic duct was visualized in most patients after CVS and FC, followed by the common bile duct and the common hepatic duct. Conversely, even after using CVS and FC, the visualization of the confluence of the cystic and common hepatic ducts remained less likely and challenging in both groups (57.3% in mild patients vs. 33.3% in moderate patients; *p* = 0.022). Background liver fluorescence disturbance was observed equally in both patient groups (6–11%), but it did not reach statistical significance. The median operative time was 60 ± 25 min in patients with mild acute cholecystitis compared to 85 ± 37 min in patients suffering from moderate acute cholecystitis (*p* < 0.001). No postoperative complications or biliovascular injuries were observed. **Conclusions:** FC is a convenient, safe, and efficacious procedure for attaining CVS principles and identifying the EHBD anatomy in most patients. The procedure showed superior results in mild acute cholecystitis patients in comparison to moderate acute cholecystitis patients.

## 1. Introduction

Gallstone disease is among the most frequently diagnosed gastrointestinal disorders, with an estimated global prevalence of 9 to 20% [1,2,3]. The prevalence of the disease varies according to gender and ethnicity, with a greater predisposition among women and an inverse correlation with age [2,3]. A significant proportion of those affected, approximately one-fifth, are reported to develop complications associated with the stones over the course of their lifetime [3]. One of the most often observed gallstone-related complications is acute cholecystitis, or inflammation of the gallbladder. While acalculous cholecystitis represents approximately 10% of all acute cholecystitis cases, it is predominantly ischemic and associated with an elevated risk of necrosis and perforation [4].

Regardless of the underlying etiology, the gold standard for the treatment of acute cholecystitis is a laparoscopic cholecystectomy (LC). Since the early 1990s, laparoscopic surgery has largely replaced the traditional open cholecystectomy, becoming the treatment of choice for gallbladder disease [5]. The rates of LCs vary considerably across Europe, with data indicating a range from 62 surgeries in Norway to 246 surgeries in Germany per 100,000 inhabitants annually [3]. Although generally considered relatively safe, the procedure has an approximate mortality rate of 0.3 to 0.5% [3]. The incidence of complications remains relatively high, with the probability of minor complications ranging from 2.6 to 5.5% and the risk of major complications ranging from 5.6 to 8.9% [6].

The most significant complication of the procedure is an iatrogenic injury to the bile duct, which typically necessitates additional surgical intervention for repair [7,8]. Bile duct injury is estimated to occur in 0.2% to 1.5% of all cholecystectomies [3,9]. In most cases, an erroneous interpretation of the biliary anatomy is the fundamental trigger for the injury [8]. Such errors are primarily the result of the presence of complex or unclear anatomic morphologies and dense adhesions. The heightened degree of inflammation and fibrosis along the dissection planes, as well as adhesions from prior surgeries, represent additional contributing factors that can increase the likelihood of a bile duct injury, conversion to open surgery, postoperative complications, and the need for subsequent surgical interventions [8].

To mitigate the risk of such iatrogenic complications, the critical view of safety (CVS) technique was introduced and has since become a widely utilized method in laparoscopic cholecystectomies. The CVS method involves removing adipose and fibrous tissue from the Calot’s triangle, isolating the inferior aspect of the gallbladder from the cystic plane, and visualizing at least two structures as they enter the gallbladder. This allows for an easier and clearer identification of the cystic duct and cystic artery, which is crucial for ensuring the safety and efficacy of the surgery [8]. Moreover, the recent advancements in intraoperative imaging using indocyanine green fluorescence (ICG) imaging have demonstrated the value of this modality in identifying vital structures during a laparoscopic cholecystectomy.

ICG imaging has been documented as a straightforward, viable, secure, and cost-effective procedure that enables surgeons to map the components of the biliary tract system in real time through biliary fluorescence imaging, thereby providing an enhanced, uninterrupted, and augmented view of the anatomy [10,11,12]. ICG is generally well-tolerated by patients, with a low rate of allergic reactions and other severe reactions [13]. Post-injection, intravenous ICG binds to plasma albumin and is exclusively excreted from the hepatocytes to the bile, making it an ideal dye for use in the fluorescence imaging of the biliary tree [14]. It has been observed that the use of ICG during LC results in a reduction in the surgical time and a lower risk of complications such as blood loss and bile duct injury, while exhibiting a superior success rate of biliary tract imaging compared to traditional modalities [11,12]. Hence, the aim of this study was to evaluate the effectiveness of ICG fluorescence cholangiography (FC) for the detection of the biliary anatomy in urgently admitted patients presenting with mild or moderate acute cholecystitis.

## 2. Methods

The present single-center, prospective, and comparative study was conducted from October 2021 to April 2024 at the General and Emergency Surgery Department of Riga East Clinical University Hospital (RAKUS), Riga, Latvia. RAKUS is one of the largest tertiary care hospitals in the country, offering both elective and emergency care, with nearly 10,000 surgical procedures performed annually in our clinic. The study protocol adhered to the principles established in the Helsinki Declaration of 2008, as revised in 2013, and received approval from the Medical and Biomedical Research Ethics Committee of the Riga East University Hospital Support Foundation (No. 9-A/20, dated 6 August 2020). All patients provided written informed consent to participate in the study.

### 2.1. Patient Selection

The study population comprised adult patients (aged 18 to 90 years) who met the criteria for mild or moderate acute cholecystitis, as recommended by the Tokyo guidelines, 2018 (Table 1) [15]. The included patients were admitted through the emergency department and prepared for an early LC (defined as within 72 h according to the Tokyo Guidelines) the next morning. The patients received ICG the previous evening. The diagnosis was based on clinical assessments by trained surgeons, combined with laboratory findings and imaging modalities such as a transabdominal ultrasound. Patients with complicated gallstone disease, allergies, or a known adverse reaction to iodine, an abnormal thyroid metabolism, thyroid malignancy, severe coagulopathy, suspected gallbladder malignancy, or contraindications to laparoscopic surgery or general anesthesia were excluded from the study. Patients who refused to provide or withdrew consent were also not included.

### 2.2. Surgical Procedure

Laparoscopic cholecystectomy with FC under general anesthesia was performed for the included patients. A dose of 12.5 mg of ICG dye with 10 cc of saline was administered intravenously 12 h prior to the surgical procedure. The surgeries were performed by a team of 2–3 certified hepato-pancreato-biliary (HPB) surgeons with expertise and confidence in laparoscopic ICG visualization. The conventional four-trocar approach was used, with the trocars placed at the 12 mm mark in the epigastrium proprium, the 10 mm mark in the umbo, and two lateral 5 mm marks on the right side of the abdomen.

Once the pneumoperitoneum had been established, the gallbladder was retracted cranially. Intraoperative scorings were performed both before and after the dissection using the Systems Green ICG/near-infrared range fluorescence imaging platform (Richard Wolf GmbH, Knittlingen, Germany). Hemoclips were used for both the cystic duct and the cystic artery. The surgical procedures were video-documented using an endoscopic camera inserted via the umbilical trocar. All patients, regardless of the surgical approach employed, received standard care in accordance with local and international guidelines, including the administration of antibacterial agents and pain management medications. A comprehensive assessment of complications and symptomatic presentation was conducted for all patients prior to their discharge from the hospital.

### 2.3. Assessment of CVS Principles

**Figure 1:**
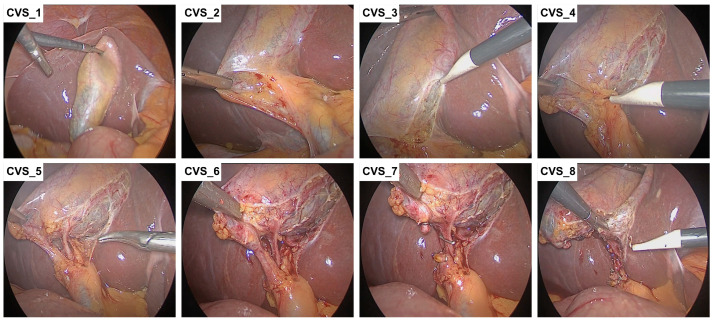
Pictorial representation of the eight steps performed as part of the critical view of safety (CVS) principles in a mild acute cholecystitis patient undergoing a laparoscopic cholecystectomy. For a detailed description of each step, refer to Table 1.

The laparoscopic cholecystectomies were performed in accordance with the CVS principles [16] following the eight-step protocol (Figure 1; Table 2). The steps were performed in a consecutive manner. The number of steps performed during the surgery was assessed before and after FC.

### 2.4. Intraoperative Assessment

Prior to the dissection of the visceral peritoneum, the visualization quality of the extrahepatic bile duct (EHBD) structures was evaluated using both the white light mode (WL) and FC modes. For the FC mode, the imaging platform detected fluorescence in the wavelength of 800–850 nm (light source: LEDgreen, Richard Wolf GmbH, Germany), where a single toggle switch enabled the transition between the WL and FC overlay modes. The EHBD structures were then reassessed under both WL and FC modes following the complete dissection of the cystic duct and the cystic artery. The FC visualization quality was assessed using a Likert scale, with 1 indicating poor quality of the bile duct visualization and 5 indicating excellent visual quality [17].

A four-point scale was employed to assess the extent to which fluorescence imaging was perceived to be helpful in each case, with 0 indicating no helpfulness and 3 signifying a high level of helpfulness. Additionally, the disturbance score was used to evaluate the extent to which background fluorescence from the liver (liver-to-duct contrast when using the ICG mode) interfered with the visualization of structures. This was achieved using a Likert-point system, with 0 indicating no disturbance and 4 indicating an extreme disturbance that made it nearly impossible to correctly visualize the biliary structures [17].

The veracity of the assigned scores was corroborated, both intraoperatively and postoperatively, through a video review, and was assessed by a single certified surgeon. The scoring scales demonstrated 100% concordance when comparing the intraoperative and postoperative scoring.

### 2.5. Study Outcomes

The perioperative data were prospectively collected by certified surgeons from the medical records and patient examinations. The curated dataset comprised the primary outcomes, including the visualization rates of bile ducts and the quality of the visualization. The secondary outcomes were bile duct injuries, the operation time, the postoperative length of the hospital stay, and postoperative complications, assessed according to the Clavien Dindo classification.

### 2.6. Statistical Analysis

A sample size estimation was not conducted due to the use of a purposive convenience sampling method for the screening and selection of patients. Continuous numerical data were checked for normality using the Shapiro–Wilk test and Q-Q plots. The data were found to follow a non-Gaussian distribution, and hence, non-parametric tests were used for comparison. For categorical independent variables, the chi-square test or Fisher’s exact test was used for association testing. For categorical dependent variables, McNemar’s test was used for 2 × 2 cross-tabulations. The Mann–Whitney U test was used to assess the differences in the distribution of numerical variables. A two-tailed *p* < 0.05 was considered statistically significant. The data analyses were performed using MS Excel 365 for Windows 11 and IBM SPSS v29.0.0 (IBM Corp, Armonk, NY, USA) for Windows 11.

## 3. Results

Our study cohort consisted of 108 patients, with 75 (69%) presenting with mild acute cholecystitis and 33 (31%) presenting with moderate acute cholecystitis. There were notable differences between the two groups in terms of their age and gender. The patients with mild acute cholecystitis exhibited a younger demographic and were predominantly female, while the patients with moderate acute cholecystitis demonstrated a more advanced age and a male predominance. Upon admission, inflammatory marker levels, such as the white blood cell count (WBC) and C-reactive protein (CRP), were elevated in both groups, with higher values observed in patients with moderate acute cholecystitis (Table 3).

### 3.1. Intraoperative Findings

Patients with mild acute cholecystitis had a significantly lower rate of peri-vesical infiltration (20% vs. 67%) and gallbladder empyema (13% vs. 55%) in comparison with the moderate acute cholecystitis patients (Table 4).

### 3.2. Number of CVS Steps Performed

To avoid bile duct injuries, it is crucial to perform at least the first four steps of the CVS principles, including the dissection of fat and fibrous tissues out of Calot’s triangle, the separation of the lowest part of the gallbladder, and the identification of two structures entering the gallbladder (cystic duct and artery). Among our cohort, more than four CVS steps were performed in 101 patients (93.5%). Fewer than four CVS steps were performed only in seven patients—three (2.5%) patients with mild acute cholecystitis and four (4%) patients with moderate acute cholecystitis (Table 5). The distribution of the number of CVS steps performed was found to be statistically significant between the mild and moderate acute cholecystitis patients, with the trend favoring mild acute cholecystitis patients.

Accordingly, we observed that performing more than four CVS steps significantly reduced the median operation time among patients with moderate acute cholecystitis (Table 6). Among mild acute cholecystitis patients, although the median time increased by 10 min when performing more than four CVS steps, the difference was not statistically significant (*p* = 0.106).

### 3.3. Primary Outcomes: Visualization of Bile Ducts Using FC

**Figure 2:**
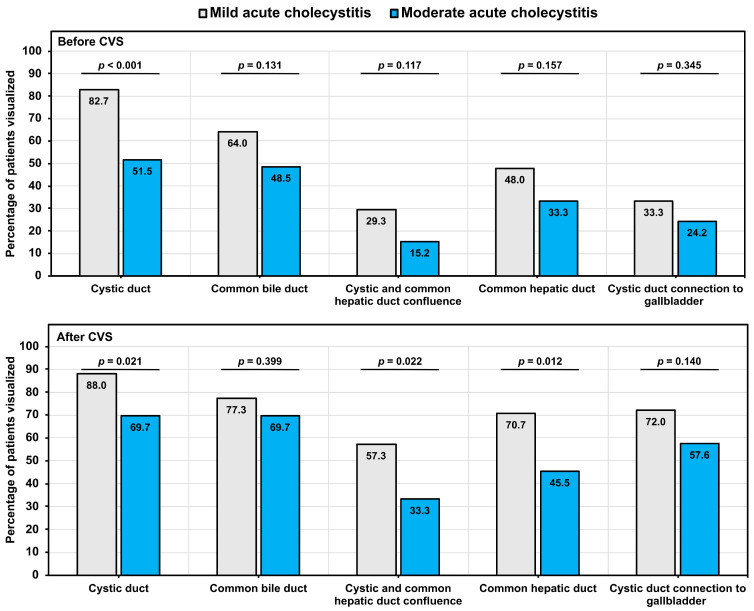
Effectiveness of CVS principles for the visualization of bile duct structures. The percentages are calculated within different groups of acute cholecystitis. The *p*-values were derived from the chi-square test.

**Figure 3:**
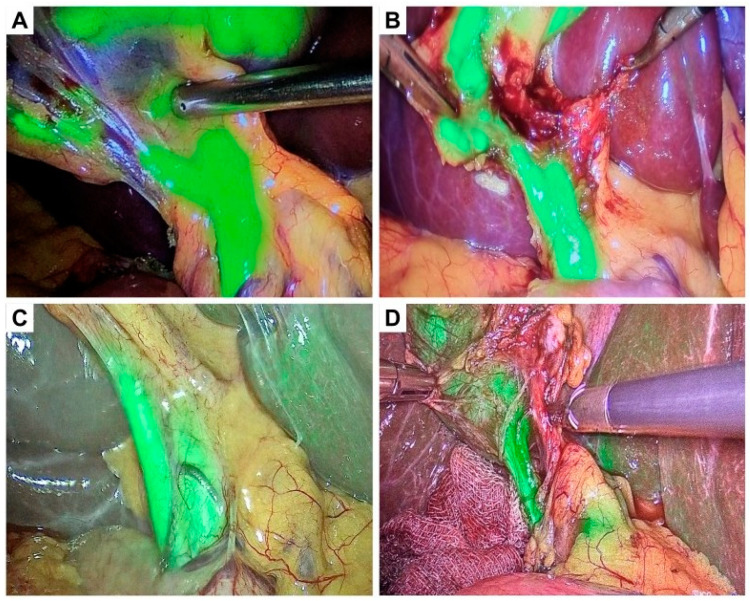
Representational intra-operative pictures before and after CVS. Green fluorescence from the ICG dye was visualized using the fluorescence cholangiography mode. The visualization of the cystic duct, the common hepatic duct, the common bile duct, and the confluence of the cystic duct and common hepatic ducts (**A**) before CVS and (**B**) after CVS in a patient with mild acute cholecystitis. The visualization of the cystic duct, the common hepatic duct, and the confluence (**C**) before CVS and (**D**) after CVS in a patient with moderate acute cholecystitis.

Using the FC mode, it was observed that the rates of visualization were higher among patients with mild acute cholecystitis in comparison to those with moderate acute cholecystitis (Figure 2 and Figure 3). Notable discrepancies in the visualization rates of the cystic duct were evident between patients with mild and moderate acute cholecystitis, both prior to (82.7% vs. 51.5%) and following (88.0% vs. 69.7%) CVS.

Moreover, the rate of visualization after CVS demonstrated significant differences in the proportional distribution of patients with mild vs. moderate acute cholecystitis for the confluence of the cystic and common hepatic ducts (57.3% vs. 33.3%) and the common hepatic duct (70.7% vs. 45.5%).

**Figure 4:**
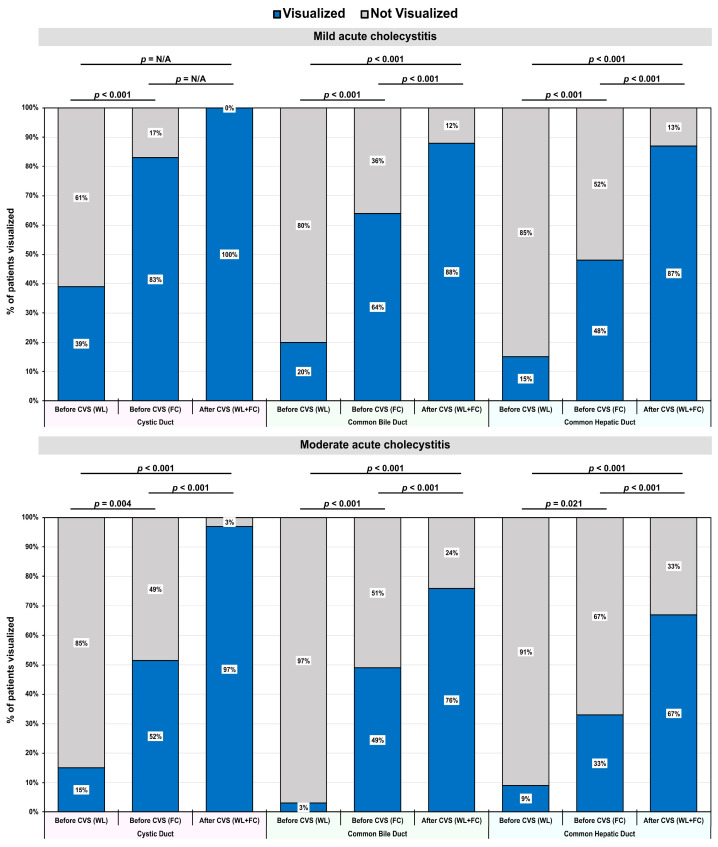
Effectiveness of CVS and FC for the improvement of the visualization of bile duct structures. The percentage of the patients visualized, stratified by the severity of the acute cholecystitis before CVS using white light (WL), before CVS using fluorescence cholangiography (FC), and after CVS using both modes (WL + FC). The percentages were calculated within different groups of acute cholecystitis. The *p*-values were derived from McNemar’s test.

Both in patients with mild and moderate acute cholecystitis, there was a notable enhancement in the rate of visualization of the cystic duct when comparing the WL and FC modes prior to CVS (Figure 4). The rate of visualization of the cystic duct in both groups approached 100% following CVS with both WL and FC modes. Regarding the rate of visualization of the common bile duct, a significant improvement was observed in both patient groups when comparing the WL mode with the FC mode before CVS. Significant differences in the visualization rates of bile ducts were observed when comparing before and after the achievement of the CVS principles with the WL mode and WL + FC modes, respectively. A similar tendency was observed when visualizing the common hepatic duct.

### 3.4. Usefulness of ICG for Visualization of Bile Ducts

There was a statistically significant association between the severity of acute cholecystitis and the usefulness of ICG for the visualization of extrahepatic bile ducts (Table 7). ICG was helpful in the visualization of the cystic duct in 83% of mildly severe patients compared with 49% of moderately severe patients (*p* < 0.001). Similarly, ICG was helpful in aiding the visualization of the common hepatic duct in 49% of mildly severe patients compared with 27% of moderately severe patients (*p* = 0.033).

It is interesting to note that, for moderate acute cholecystitis patients, ICG demonstrated a notably poor effectiveness across all structures. Since, during surgery, moderate cholecystitis cases typically require the more precise identification of the biliary tree compared to mild cases, ICG’s effectiveness in moderate acute cholecystitis patients needs to be assessed further.

### 3.5. Assessment of Background Liver Fluorescence

**Figure 5:**
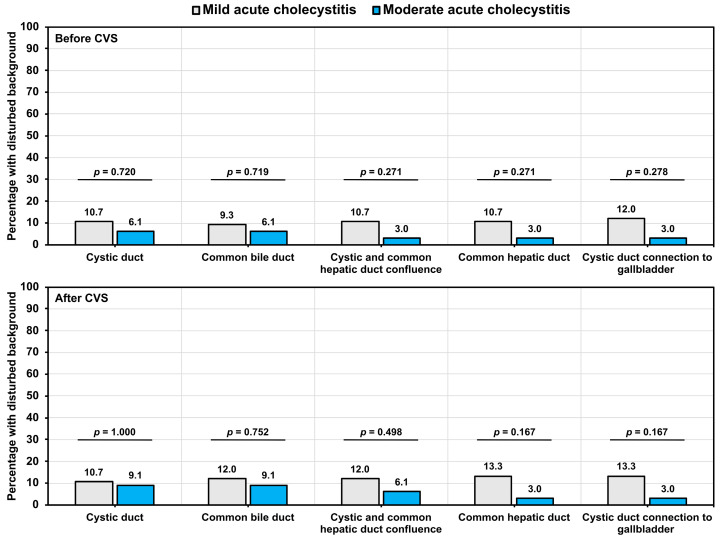
The percentage of patients with a disturbed background, stratified based on the severity of acute cholecystitis before and after CVS. The percentages were calculated within different groups of acute cholecystitis. The *p*-values were derived from Fisher’s exact test.

The background liver fluorescence was assessed before and after the CVS principles. Although a higher rate of disturbance in the background fluorescence was observed for cystic ducts among mild acute cholecystitis patients when compared with moderate acute cholecystitis patients, both before (10.7% vs. 6.1%; *p* = 0.720) and after CVS (10.7% vs. 9.1%; *p* = 1.000), the difference was not statistically significant (Figure 5).

### 3.6. Secondary Outcomes

The operation time was significantly shorter in mild acute cholecystitis patients, with no patient requiring conversion to an open approach. However, three patients with moderate acute cholecystitis required conversion to an open approach due to the presence of severe peri-vesicular infiltration. The WBC count and CRP at discharge were significantly higher in patients with moderate acute cholecystitis (Table 8). All the patients were classified as Grade I Clavien Dindo in terms of postoperative complications, with no cases of dropout. No differences in the length of the hospital stay were observed between the two groups. No iatrogenic bile duct injuries were reported in either group.

## 4. Discussion

FC has gained considerable traction in recent years and has been demonstrated to enhance the visualization rate of EHBD structures while concurrently reducing the intraoperative risk of bile duct injuries [18,19,20,21]. Primarily, the enhanced visualization facilitates the navigation of surrounding structures and the identification of vital vasculature and bile ducts that are challenging to discern in patients presenting with acute cholecystitis. Advanced peri-vesical inflammation, peri-cholecystic adhesions, a high BMI, and anatomical variations often present challenges to the precise identification of the EHBD structures, even for experienced surgeons, contributing to an increased risk of iatrogenic biliovascular injuries [22,23].

Such injuries have the potential to lead to late complications such as anastomotic strictures, recurrent cholangitis, and secondary biliary cirrhosis, necessitating prolonged hospitalizations and repeated surgical interventions [22,24,25]. Our study showed that the frequency of bile duct visualization using FC was higher in patients with mild acute cholecystitis in comparison to patients with moderate acute cholecystitis.

These discrepancies may be attributed to the inflammation of the gallbladder and the surrounding tissues [26,27], with higher rates of gallbladder inflammation and empyema noted in patients with moderate acute cholecystitis. The degree of intra-abdominal fatty tissue and adiposity in the hepatoduodenal ligament are other factors that can affect the quality of the fluorescence effect [28]. The visualization rate of EHBD structures using FC may be attributed to the limited tissue penetration of the ICG fluorescent light, which has been estimated to be approximately 5–10 mm [29,30]. In patients with substantial peritoneal adiposity or peritoneal scarring due to inflammation, the light may not effectively penetrate the tissues [30]. The stepwise usage of the CVS protocol under FC effectively allows a surgeon to visualize the bile duct anatomy and complete the dissection of the cystic duct from the surrounding tissues.

Additionally, the visualization of the bile ducts can be altered not only by localized inflammation and peritoneal adiposity, but also by the inflammation of liver. An animal study that mimicked acute and chronic liver injury in rats demonstrated that the internalization of the ICG dye by hepatocytes was markedly compromised, resulting in the incomplete visualization of the liver lobes and a significant reduction in the spectral intensities for yellow and green hues [31]. It seems reasonable to hypothesize that a comparable process may occur in humans, whereby a hepatocytic injury resulting from inflammatory infiltration in the context of acute cholecystitis could potentially impair ICG metabolism and the subsequent secretion into the bile [32,33]. Further research is required to investigate this relationship between the ICG plasma disappearance rate (ICG-PDR) and the EHBD visualization rate and quality.

A higher rate of visualization of extrahepatic bile ducts with FC has been documented in the literature than observed in our study [17,20,34,35]. Similar observations have been reported in patients with acute cholecystitis who underwent FC-assisted robotic surgery. When using the FC mode during the robotic cholecystectomy, a rise in the rate of the identification of the cystic duct, common bile duct, and common hepatic duct was reported, both before and after Calot’s triangle dissection [36,37,38]. For example, the FALCON trial showed a higher rate of CBD detection. However, it is noteworthy that the FALCON trial patients underwent an elective LC [39], in contrast to our study, where the patients underwent an urgent LC.

Furthermore, these studies did not stratify patients according to the severity of their acute cholecystitis, which could explain the differences. Notable discrepancies were also identified in the ICG administration protocols implemented across the studies, particularly with reference to the dosage, timing, and frequency of repeated doses. While our protocol involved the administration of a higher dosage (12.5 mg) than observed in other studies [12,40], previous studies have opted to administer the contrast agent less than an hour before the incision and/or to utilize repeated doses with the aim of improving visualization [17,20,34,35,36,37,38]. This is of particular importance, given that multivariate analyses have demonstrated that both the timing and dosage of ICG can have a significant impact on the quality of visualization [17]. Nonetheless, we do acknowledge that the administration of ICG 12 h before an LC may prove to be challenging in cases of indicated early surgery, regardless of how much time has passed since the onset of symptoms, as recommended by the Tokyo guidelines.

In our clinics, the extended CVS protocol is used, comprising of eight steps starting from visceral peritoneum dissection to gallbladder removal from the liver bed [16]. The CVS steps were performed with the help of FC, and the results showed that, in all cases, the cystic duct was successfully identified, which is a critical aspect of performing a safe cholecystectomy. Other studies have concurred with the observation that, to achieve the CVS principles, the dissection of fat and fibrous tissues out of Calot’s triangle, the separation of the lowest part of the gallbladder, and the identification of two structures entering the gallbladder (cystic duct and artery) are essential to avoid bile duct injuries [41]. Clearly, FC enhances the visualization of extrahepatic bile ducts, allowing CVS to be safely achieved, even in patients with moderately severe acute cholecystitis.

All operations in our study were performed by 2–3 trained hepato-pancreato-biliary surgeons that strictly followed the extended CVS principles. The extended CVS protocol began with the preoperative administration of ICG and concluded with the evaluation of the liver bed to exclude bile leaks [42]. The extended CVS principles, together with strict ICG administration protocols, can be considered as strengths of our study. It is noteworthy that, in moderate acute cholecystitis patients, the achievement of the CVS could be counter-indicated, as complications can occur during the dissection. In our study as well, we achieved a few CVS steps only in this group of patients to lower the risk of biliovascular injuries [43].

It is important to note that our study is subject to certain limitations. The study population was rather limited, and the obtained results require further validation and replication in larger, multi-center cohorts. Our study groups were disproportionately distributed in a 2:1 ratio (mild/moderate acute cholecystitis patients), a reflection of the general trend of patient hospitalization patterns encountered in our department. Furthermore, an overall small sample size limited the possible generalizability of the *p*-values so obtained in our analyses. Similarly, diversifying the demographic pool of patients in terms of gender, age groups, and BMI could further refine the results. A video-archive review by a single certified surgeon also leaves room for subjectivity and the need for inter-rater agreement in future studies. Nonetheless, our study shows that FC improves the visualization of EHBD structures in most patients with mild or moderate acute cholecystitis when a stepwise extended CVS protocol is used. Both CVS and FC help surgeons to perform a “*safe cholecystectomy*”.

## 5. Conclusions

Fluorescence cholangiography (FC) was found to be an effective method for the visualization of extrahepatic bile ducts during urgent laparoscopic cholecystectomy, especially for patients with mild acute cholecystitis. However, our findings also highlight the significant limitations of FC in moderate acute cholecystitis patients. In moderate acute cholecystitis patients, we experienced lower visualization rates of critical structures such as cystic and common hepatic ducts, longer operative times, and procedural challenges, even after adherence to the critical view of safety (CVS) protocol. Factors such as increased peri-vesical inflammation, gallbladder empyema, and tissue adiposity likely contributed to the lower effectiveness of FC in such patients. While no iatrogenic biliovascular injuries were observed, the findings highlight the need to optimize the FC protocols to improve their efficacy in acute cholecystitis patients. Future research should validate our observations in larger, multi-center cohorts to ensure patient safety and procedural success.

## Figures and Tables

**Table 1:** Severity classification for acute cholecystitis patients based on the Tokyo guidelines, 2018.

Severity Grade	Diagnostic Criteria
Acute Cholecystitis	*Definite diagnosis*: one item in A + one item in B + C. *Suspected diagnosis*: one item in A + one item in B. A. Local signs of inflammation—(1) Murphy’s sign or (2) right upper quadrant mass/pain/tenderness. B. Systemic signs of inflammation—(1) Fever, (2) elevated CRP, or (3) elevated WBC count. C. Imaging findings—Imaging findings characteristic of acute cholecystitis.
Mild (Grade I)	Acute cholecystitis patient meeting any of the following criteria: Symptoms that do not meet the criteria of Grade III or Grade II acute cholecystitis. Healthy patient with no organ dysfunction and mild inflammatory changes.
Moderate (Grade II)	Acute cholecystitis patient presenting with any of the following symptoms: Leukocytosis (>18,000/mm^3^). Palpable tender mass in the right upper abdominal quadrant. Duration of complaints > 72 h. Marked local inflammation (gangrenous cholecystitis, pericholecystic abscess, hepatic abscess, biliary peritonitis, emphysematous cholecystitis).
Severe (Grade III)	Acute cholecystitis patient presenting with dysfunction of any of the following organs/organ systems: Cardiovascular dysfunction: hypotension requiring treatment with dopamine ≥ 5 μg/kg/min, or any dose of norepinephrine. Neurological dysfunction: decreased level of consciousness. Respiratory dysfunction: PaO_2_/FiO_2_ ratio < 300. Renal dysfunction: oliguria, creatinine > 2.0 mg/dL. Hepatic dysfunction: PT-INR > 1.5. Hematological dysfunction: platelet count < 100,000/mm^3^.

**Table 2:** Overview of the critical view of safety (CVS) steps.

CVS Step	CVS Step Description
I	Cranial retraction of the *fundus* part of the gallbladder.
II	Lateral retraction of the *infundibulum* part of the gallbladder.
III	Dissection of the visceral peritoneum with electrocoagulation, either laterally or medially, from the *infundibulum* part, ascending to the *fundus* part of the gallbladder.
IV	Dissection of the medial adipose tissue of the gallbladder with electrocoagulation, visualization and release of the cystic duct, and visualization of its entry into the gallbladder.
V	Total dissection of adipose tissue and formation of a “critical safety triangle” by separating cystic duct and cystic artery.
VI	Dissection of the *infundibulum* part of the gallbladder from adipose tissue and mobilization in the anterior/posterior parts, creating the “Calot’s triangle”. Visualization of the margin of the liver.
VII	Clipping of the cystic duct (distal/proximal) from the gallbladder and its resection. Clipping of cystic artery and its resection.
VIII	Dissection of the gallbladder from the liver bed.

**Table 3:** Baseline patient characteristics.

Characteristics ^†^	Mild Acute Cholecystitis (n = 75)	Moderate Acute Cholecystitis (n = 33)	*p*-Value
Patient gender			
Male	23 (59%)	16 (41%)	*p* = 0.076 **
Female	52 (75%)	17 (25%)	
Age (years)	54 (24 to 85)	64 (24 to 86)	*p* = 0.014 *
BMI (kg/m^2^)	27.9 (7.1)	28.0 (6.6)	*p* = 0.038 *
WBC count on admission (10^9^ cells/L)	12.0 (4.0)	14.0 (5.5)	*p* < 0.001 *
CRP on admission (mg/L)	16.5 (38.5)	42.0 (67.5)	*p* < 0.001 *

^†^ Median and interquartile range (IQR) are reported. For age, median and range are reported. For gender, number of patients and % are reported. * Mann–Whitney U test; ** chi-square test.

**Table 4:** Summary of intraoperative findings.

Characteristics ^†^	Mild Acute Cholecystitis (n = 75)	Moderate Acute Cholecystitis (n = 33)	*p*-Value
Intraoperative detection of peri-vesicular infiltration			
Yes	15 (20%)	22 (67%)	*p* < 0.001 *
No	60 (80%)	11 (33%)	
Gallbladder empyema			
Yes	10 (13%)	18 (55%)	*p* < 0.001 *
No	65 (87%)	15 (45%)	

^†^ For both categorical variables, number of patients and % are reported. * Chi-square test.

**Table 5:** Distribution of patients (n, %) based on number of CVS steps performed.

No. of CVS Steps Performed	Mild Acute Cholecystitis	Moderate Acute Cholecystitis	*p*-Value *
0 steps	0 (0%)	1 (3%)	0.002
1 step	0 (0%)	1 (3%)	
2 steps	1 (1%)	0 (0%)	
3 steps	0 (0%)	0 (0%)	
4 steps	2 (3%)	2 (6%)	
5 steps	5 (7%)	6 (18%)	
6 steps	16 (21%)	9 (27%)	
7 steps	8 (11%)	5 (16%)	
8 steps	43 (57%)	9 (27%)	

* *p*-value derived from the Mann–Whitney test.

**Table 6:** Operation time in minutes based on number of CVS steps performed.

No. of CVS Steps Performed	Operation Time (min)				Mann–Whitney *p*-Value
	Mild Acute Cholecystitis		Moderate Acute Cholecystitis		
	Median	IQR	Median	IQR	
0–4 steps (n = 7)	45.0	-	105.0	47.5	0.057
5–8 steps (n = 101)	55.0	25.0	75.0	30.0	<0.001
**Mann–Whitney *p*-value**	0.106		0.009		-

**Table 7:** Assessment of usefulness of ICG (presented as n, %).

Structure (n = 108)	Severity of Acute Cholecystitis	Usefulness of ICG for CVS		Chi-Square Test *p*-Value
		Not Helpful	Helpful	
Cystic duct	Mild (n = 75)	13 (17%)	62 (83%)	*p* < 0.001
	Moderate (n = 33)	17 (52%)	16 (49%)	
Common bile duct	Mild (n = 75)	27 (36%)	48 (64%)	*p* = 0.072
	Moderate (n = 33)	18 (55%)	15 (45%)	
Cystic and common hepatic duct confluence	Mild (n = 75)	53 (71%)	22 (29%)	*p* = 0.054
	Moderate (n = 33)	29 (88%)	4 (12%)	
Common hepatic duct	Mild (n = 75)	38 (51%)	37 (49%)	*p* = 0.033
	Moderate (n = 33)	24 (73%)	9 (27%)	
Cystic duct connection to gallbladder	Mild (n = 75)	52 (69%)	23 (31%)	*p* = 0.312
	Moderate (n = 33)	26 (79%)	7 (21%)	

**Table 8:** Overview of surgical outcomes.

Characteristics ^†^	Mild Acute Cholecystitis (n = 75)	Moderate Acute Cholecystitis (n = 33)	*p*-Value
Operation time (min)	60.0 (25.0)	85.0 (37.5)	*p* < 0.001 *
Conversion to open approach			
Yes	0 (0%)	3 (9%)	*p* = 0.027 **
No	75 (100%)	30 (91%)	
WBC count on discharge (10^9^ cells/L)	7.0 (3.0)	10.0 (4.5)	*p* = 0.002 *
CRP on discharge (mg/L)	30.0 (53.0)	60.0 (105.5)	*p* = 0.002 *
Hospitalization length (days)	6.0 (3.0)	6.0 (4.5)	*p* = 0.437 *
Length of postoperative stay (days)	2.0 (2.0)	3.0 (2.0)	*p* < 0.001 *
Biliovascular injuries (No. of patients)	0 (0)	0 (0)	-
Mortality (No. of patients)	0 (0)	0 (0)	-

^†^ Median and interquartile range (IQR) are reported. For categorical variables, raw count (%) is reported. * Mann–Whitney U test; ** Fisher’s exact test due to violation of assumptions for chi-square test.

## Data Availability

The underlying dataset is available for non-commercial purposes from the corresponding author upon reasonable request.
